# Boosting alternating decision trees modeling of disease trait information

**DOI:** 10.1186/1471-2156-6-S1-S132

**Published:** 2005-12-30

**Authors:** Kuang-Yu Liu, Jennifer Lin, Xiaobo Zhou, Stephen TC Wong

**Affiliations:** 1 HCNR Center for Bioinformatics, Brigham and Women's Hospital, Harvard Medical School, Boston, MA 02215 USA; 2 Department of Radiology, Brigham and Women's Hospital, Harvard Medical School, Boston, MA 02215 USA; 3 Division of Preventive Medicine, Brigham and Women's Hospital, Harvard Medical School, Boston, MA 02215 USA

## Abstract

We applied the alternating decision trees (ADTrees) method to the last 3 replicates from the Aipotu, Danacca, Karangar, and NYC populations in the Problem 2 simulated Genetic Analysis Workshop dataset. Using information from the 12 binary phenotypes and sex as input and Kofendrerd Personality Disorder disease status as the outcome of ADTrees-based classifiers, we obtained a new quantitative trait based on average prediction scores, which was then used for genome-wide quantitative trait linkage (QTL) analysis. ADTrees are machine learning methods that combine boosting and decision trees algorithms to generate smaller and easier-to-interpret classification rules. In this application, we compared four modeling strategies from the combinations of two boosting iterations (log or exponential loss functions) coupled with two choices of tree generation types (a full alternating decision tree or a classic boosting decision tree). These four different strategies were applied to the founders in each population to construct four classifiers, which were then applied to each study participant. To compute average prediction score for each subject with a specific trait profile, such a process was repeated with 10 runs of 10-fold cross validation, and standardized prediction scores obtained from the 10 runs were averaged and used in subsequent expectation-maximization Haseman-Elston QTL analyses (implemented in GENEHUNTER) with the approximate 900 SNPs in Hardy-Weinberg equilibrium provided for each population. Our QTL analyses on the basis of four models (a full alternating decision tree and a classic boosting decision tree paired with either log or exponential loss function) detected evidence for linkage (Z ≥ 1.96, *p *< 0.01) on chromosomes 1, 3, 5, and 9. Moreover, using average iteration and abundance scores for the 12 phenotypes and sex as their relevancy measurements, we found all relevant phenotypes for all four populations except phenotype b for the Karangar population, with suggested subgroup structure consistent with latent traits used in the model. In conclusion, our findings suggest that the ADTrees method may offer a more accurate representation of the disease status that allows for better detection of linkage evidence.

## Background

Alternating decision trees (ADTrees) are machine learning methods combining boosting and decision trees algorithms to generate classification rules [1]. Traditional boosting decision tree algorithms such as CART [2] and C4.5 [3] have been successful in generating classifiers but at the cost of creating complicated decision-tree structures. Such structures often represent convoluted decision rules that are hard to interpret [1]. In contrast, ADTrees generate simpler decision-tree structures and easier-to-interpret classification rules while their use of AdaBoost algorithm during training require fewer iteration cycles [1].

ADTrees, a natural extension of both voted-stumps and decision trees, consist of alternating layers of prediction and decision nodes [1]. The structure of an ADTree represents decision paths; when a path reaches a decision node, it continues with the specific offspring node that corresponds to the decision outcome as in standard decision tree. On the other hand, when a path reaches a prediction node, the path continues with all of the offspring nodes. Thus the classification rule that it represents is basically a weighted majority vote over base prediction rules.

**Figure 1:**
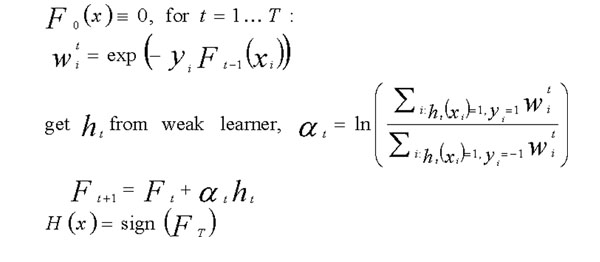
The AdaBoost algorithm.

Boosting is a general and effective method of combining moderately successful rules to produce very accurate prediction. As outlined in Figure 1, each weak prediction rule in the AdaBoost algorithm [4,5] is associated with a prediction node. At each boosting iteration step, *t*, a decision node, together with its two prediction nodes, is introduced. For full ADTrees, the decision node may be attached to any previous prediction node, leaf nodes or otherwise, including the root prediction node. Each prediction node is associated with a weight, *α*, which represents its contribution to the final prediction score, *F*(*x*), for every path that reaches it. Hence the contribution of each decision node may be understood in isolation, and summing the individual contribution gives rise to the final prediction and classification.

In this study, we present the results of ADTrees in conjunction with genome-wide quantitative trait linkage (QTL) scans. Three quantitative measurements, including prediction, iteration, and abundance scores, were obtained from the ADTree-based classifiers using information from the 12 phenotypes and sex. With the help of the quantitative information, we aimed to gain a better understanding of the relationship between disease phenotypes and affection status as well as the risk profiles for each individual. The prediction scores, serving as a composite view of disease status, were then used to carry out genome-scan linkage analysis.

## Methods

The Problem 2 simulated data were used in this study and all analyses were performed without knowledge of the answers except the final comparison with disease models and regions of real linkage. The datasets comprised four populations, Aipotu, Danacca, Karangar, and NYC. Within each population, we arbitrarily selected the last three replicates out of the total 100 replicates available to the Genetic Analysis Workshop 14 (GAW14) participants.

We used ADTrees decision-trees with AdaBoost boosting algorithm implemented in MLJAVA [6] to construct a new disease trait based on information of the features, that is, 12 phenotypes and sex. During the boosting iteration, either log or exponential loss function was used. In addition, we carried out two choices of tree generation types: a full alternating decision tree (full-ADT) as well as a "classic" boosting decision tree (BDT). The affection status, sex, and the 12 phenotypes were recoded as follows: the affected was coded as +1 and unaffected as -1; the male was coded as +1, and female, -1; each trait was coded as +1 when present, and -1 when absent. The affection status was used as the classification label while sex and the 12 phenotypes were the features that the classifiers were trained to recognize.

We chose founders in each population to construct the classifiers. The classifiers for each population were trained and tested by a total of 10 runs of 10-fold cross validation. Within each run, one-tenth of the founders in each population were reserved as a test set and the rest were used as a training set.

**Figure 2:**
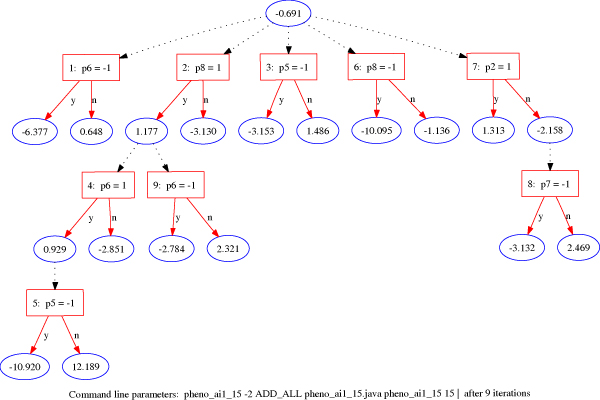
**A full alternating decision tree (full-ADT)**. Rectangles represent decision nodes; ellipses are prediction nodes. The number placed before each introduced feature in the rectangle is the boosting iteration step.

The prediction score of disease status for each individual was derived directly from the ADTree model. Figure 2 illustrates a typical ADTree model contains root prediction node (the top ellipse, unconditional contribution score), decision nodes (in rectangles, introduced features), prediction nodes (in ellipses, contribution scores), and decision paths (in solid lines and dashed lines, decision conditions and prediction contributions). In principle, when a path reaches a rectangle, a solid line corresponding to a decision outcome was obtained along with a specific ellipse. On the other hand, when a path reached an ellipse, the path continues with all of the dashed lines and considers multiple paths reaching all the subsequent rectangles. The prediction score for a classification tree model is the sum of all the contribution scores reached along the paths. As shown in Figure 2, an individual with phenotypes b, e, f, h present and the remaining phenotypes absent would have a prediction score of 18.24. Recall that the affection status was used as the classification label: +1 being affected and -1 unaffected. Because the prediction scores may be obtained by various number of decision nodes (or introduced features), we standardized the prediction scores by dividing by the maximum (if the prediction scores > 0) or the minimum (if the prediction scores < 0) prediction score for that run. Thus, positive standardized prediction scores corresponded to being more likely to be affected (≤ 1 and > 0), and negative scores, unaffected (≥ -1 and < 0). The final prediction score for each individual was obtained by averaging all the standardized prediction scores over 10 runs of 10-fold cross validation.

The iteration score and abundance score were additional quantitative measurements estimated from the individual tree structure associated with a classifier built during each run [7]. The number placed before colon in the rectangle is the iteration step during the boosting in which the particular feature (i.e., 12 phenotypes or sex) was introduced; a lower iteration number associated with the introduced feature indicates a more important decision rule. For instance, phenotype h in Figure 2 was first introduced after 2 boosting iteration steps, and thus we gave Phenotype h an iteration score of 2 in this run. The abundance score was the number of occurrences in the decision trees. Because phenotype h in Figure 2 appeared twice, the abundance score for Phenotype h was assigned as 2. The iteration and abundance scores were then averaged over 10 runs of 10-fold cross validation. Hence, average iteration and abundance scores of a feature represented the measurements of its relevancy in determining disease status outcomes.

The prediction scores were used as a quantitative trait for QTL analysis in four populations using 917 single-nucleotide polymorphism (SNP) genotypes in the last 3 replicates. Prior to the analysis, Hardy-Weinberg equilibrium (HWE) tests of each population were first carried out using founder genotype information. Around 900 SNPs in HWE (900 in the Aipotu, 903 in the Danacca, 897 in the Karangar, and 904 in the NYC populations) were then retained for further analyses. Four genome scans for each population were performed using prediction scores computed from four different classification models; that is, full-ADT or BDT tree models paired with either exponential or log loss function. The expectation-maximization Haseman-Elston (EM-HE) method as implemented in GENEHUNTER [8] was used for multipoint QTL analysis. "Pairs used" option was set to "all pairs of affected/phenotyped sibs." Allele frequencies were acquired from map files.

## Results

In all four populations, the average size of iteration steps over 10 runs of 10-fold cross validation during the boosting appeared to be the smallest in the full-ADT and largest in the BDT classifiers, suggesting that the full-ADT classifier performed better than the BDT. Although the log loss function appeared to perform slightly better than the exponential loss function except in the Karangar population, the two loss functions only differed by one step. The average size of all four classification models in all four populations is presented in Table 1.

Full-ADT with either exponential or log loss function extracted all relevant phenotypes used in the disease simulation models for all four populations without any false positive through either iteration or abundance scores averaged over 10 runs of 10-fold cross validation, as shown in Table 2. On the other hand, phenotype b for the Karangar population was the sole false negative. Specifically, phenotypes b, c, d, e, f, g, and h were chosen for the Aipotu population; phenotypes b, e, f, and h were chosen for the Danacaa population; phenotypes c, d, e, f, g, and h were chosen for the Karangar population; phenotypes b, c, d, e, f, g, and h were chosen for the NYC population. In addition, the order of relevant phenotypes by average iteration and abundance scores, as shown in Table 2, indicated that the four populations were quite different from one another. Overall, BDT performed less well than full-ADT in terms of extracting relevant phenotypes.

Furthermore, iteration scores of relevant phenotypes from full-ADT suggested certain grouping structures, as shown in Table 2; phenotype b was in its own subgroup while phenotypes (e, f, h) and phenotypes (c, d, g) were in two separate subgroups, respectively. Although we did not recover the full simulated model, our observation of subgroups within relevant phenotypes was consistent with latent traits used in the model. In particular, latent trait P1 in the simulated model was made of phenotype b and phenotypes (e, f, h); latent trait P2 was made of phenotypes (e, f, h) and phenotypes (c, d, g); latent trait P3 was made of phenotype b, phenotypes (e, f, h) and phenotypes (c, d, g).

Using average prediction scores from full-ADT as a quantitative trait for QTL analysis, with either exponential or log loss function then averaged over 10 runs of 10-fold cross validation, our genome scans attained significant evidence of linkage (Z ≥ 1.96, *p *< 0.01) on chromosomes 1, 3, 5, and 9, where SNP markers were closest to disease-related loci, as shown in Table 3. For the Aipotu population, the QTL analyses detected linkage on chromosomes 1, 3, and 5, while neglecting chromosome 9. For the Danacca population, genome scans detected linkage on chromosomes 1 and 3. Using full-ADT with either exponential or log loss function, our scans detected linkage on chromosomes 3, 5, and 9 but missed chromosome 1 for the Karangar population. Our scans detected linkage on chromosomes 1, 3, and 5 but missed chromosome 9 for the NYC population. For the two "modifying" loci on chromosomes 2 and 10, we missed both for the Aipotu, Karangar, and NYC populations. Our genome scans detected false-positive evidence of linkage on chromosome 8 for the Aipotu, chromosome 10 for the Danacca, chromosome 2 for the Karangar, and chromosome 7 for the NYC populations. SNP markers with false-negative evidence of linkage are shown in italics in Table 3.

## Conclusion

In this study, we applied the ADTrees method to the Problem 2 dataset and generated a new quantitative trait for each individual based on average prediction scores using information from the 12 phenotypes and sex. In short, the full-ADT approach performed better than BDT, while the exponential and logarithmic loss functions provided essentially comparable results. Using average iteration and abundance scores for the 12 phenotypes and sex, full-ADT extracted all relevant phenotypes without any false positive for all four populations except phenotype b for the Karangar population. Furthermore, iteration scores of relevant phenotypes from full-ADT suggested a subgroup structure consistent with latent traits used in the model. The order of relevant phenotypes by average iteration and abundance scores also indicated that the four populations were quite different from one another. In the QTL analyses, these methods identified significant evidence of linkage where SNP markers were closest to disease-related loci. SNP markers with false-negative and false-positive evidence of linkage were also considered. Future QTL analysis in combination with iteration scores and abundance scores may yield even better results than traditional qualitative trait linkage through a summary of relevant traits that contribute to disease status. In summary, our findings suggest that the ADTrees method is feasible and offers the advantage of a more accurate representation of the disease status.

## Abbreviations

ADTrees: Alternating decision trees

BDT: Boosting decision tree

EM-HE: Expectation-maximization Haseman-Elston

GAW14: Genetic Analysis Workshop 14

HWE: Hardy-Weinberg equilibrium

QTL: Quantitative trait linkage

SNP: Single-nucleotide polymorphism

## Authors' contributions

K-YL conceived of the study, participated in its design, carried out ADTrees analyses, and drafted the manuscript. JL participated in its design, and carried out QTL linkage analyses. XZ was responsible for the background in machine learning methodology. STCW participated in the design of the study. All authors read and approved the final manuscript.

## Figures and Tables

**Table 1:** Averaged number of iteration steps during the boosting in the four classification models (full alternative (full-ADT) or classic boosting decision trees (BDT) paired with exponential (Exp) or log (Log) loss function in the four populations.

Model	Aipotu	Danacca	Karangar	NYC
full-ADT & exponential	10.9	9.1	11.8	12.3
full-ADT & log	10.4	7.7	12.7	11.6
BDT & exponential	13.2	12.9	10.6	14.8
BDT & log	14.2	13.9	14	13.9

**Table 2:** Relevant phenotypes in the two classification models for the four populations ordered by iteration scores and abundance scores.

Model	Aipotu	Danacca	Karangar	NYC
Iteration scores				
full-ADT & exponential	f, h, e, b, g, c, d	b, e, f, h	g, f, d, e, h, c	h, f, e, b, c, d, g
full-ADT & log	f, h, e, b, g, c, d	b, e, f, h	g, f, d, e, h, c	h, f, e, b, c, d, g

Abundance scores				
full-ADT & exponential	e, f, h, b, c, d, g	h, b, e, f	d, c, g, e, h, f	e, d, f, c, b, h, g
full-ADT & Log	e, g, h, c, f, d, b	h, e, b, f	d, g, c, h, e, f	d, c, e, b, f, g, h

**Table 3:** Haseman-Elston Z-scores on markers closest to disease-related loci for the two classification models in the four populations

Population	Model		Chromosomal regions					
			1	3	5	9	2	10

Aipotu	full-ADT & Exp	Marker	C01R0052	C03R0280	C05R0379	*C09R0765^a^*	*C02R0097*	*C10R0880*
		Position	169.60	295.89	3.07	*5.83*	*14.87*	*70.43*
		Z-score	3.13	3.41	2.96	*1.81*	*0.47*	*1.84*
	full-ADT & Log	Marker	C01R0052	C03R0280	C05R0379	*C09R0765*	*C02R0097*	*C10R0880*
		Position	169.60	295.89	3.07	*5.83*	*14.87*	*70.43*
		Z-score	3.19	3.57	2.78	*1.80*	*0.33*	*1.85*

Danacca	full-ADT & Exp	Marker	C01R0052	C03R0281	--^b^	--	--	--
		Position	169.60	298.31	--	--	--	--
		Z-score	5.62	3.16	--	--	--	--
	full-ADT & Log	Marker	C01R0052	C03R0281	--	--	--	--
		Position	169.60	298.31	--	--	--	--
		Z-score	5.66	3.55	--	--	--	--

Karangar	full-ADT & Exp	Marker	*C01R0052*	C03R0281	C05R0380	C09R0765	*C02R0097*	*C10R0880*
		Position	*169.60*	298.31	5.74	5.83	*14.87*	*70.43*
		Z-score	*1.39*	4.18	3.65	4.28	*0.03*	*0.68*
	full-ADT & Log	Marker	*C01R0052*	C03R0281	C05R0380	C09R0765	*C02R0097*	*C10R0880*
		Position	*169.60*	298.31	5.74	5.83	*14.87*	*70.43*
		Z-score	*1.41*	4.14	3.76	4.27	*0.00*	*0.61*

NYC	full-ADT & Exp	Marker	C01R0052	C03R0278	C05R0380	*C09R0765*	*C02R0097*	*C10R0880*
		Position	169.60	290.37	5.74	*5.83*	*14.87*	*70.43*
		Z-score	2.20	4.56	2.21	*1.72*	-*0.34*	-*0.92*
	full-ADT & Log	Marker	C01R0052	C03R0278	C05R0380	*C09R0765*	*C02R0097*	*C10R0880*
		Position	169.60	290.37	5.74	*5.83*	*14.87*	*70.43*
		Z-score	2.32	4.72	2.19	*1.77*	-*0.29*	-*0.88*

^a^SNP markers with false negative evidence are in italics.

^b^--, no disease related loci on the chromosome according to the disease model given by GAW14 Problem 2.
